# Early Vancomycin Concentrations and the Applications of a Pharmacokinetic Extrapolation Method to Recognize Sub-Therapeutic Outcomes

**DOI:** 10.3390/pharmacy4040037

**Published:** 2016-11-10

**Authors:** Oscar Santalo, Umima Baig, Mara Poulakos, Daniel Brown

**Affiliations:** Gregory School of Pharmacy, Palm Beach Atlantic University, West Palm Beach, FL 33401, USA; umima_baig@pba.edu (U.B.); mara_poulakos@pba.edu (M.P.); dan_brown@pba.edu (D.B.)

**Keywords:** vancomycin, therapeutic drug monitoring, trough, serum concentration, pharmacokinetic dosing

## Abstract

Vancomycin trough concentrations should be measured within 30 min of the next dose, but studies have shown that troughs are often measured too early, producing erroneous results that could lead to dosing errors. The purpose of this study was to identify the frequency of early trough measurements and to evaluate whether pharmacokinetically extrapolating mistimed concentrations may locate sub-therapeutic concentrations. Vancomycin troughs were retrospectively reviewed. For troughs ≥10 mg/L and measured >0.5 h early, the true trough was estimated using pharmacokinetic extrapolation methods to identify sub-therapeutic outcomes. Differences ≥2 mg/L between the measured and estimated true trough level was considered to have potential clinical significance. Of 143 troughs evaluated, 62 (43%) were measured too early and 48 of those troughs were ≥10 mg/L. 25% of those 48 troughs were sub-therapeutic. The potential for a difference ≥2 mg/L between the measured and estimated true trough was found to be greatest when the measured trough was ≥10 mg/L, the patient’s creatinine clearance (CrCl) was ≥60 mL/min, and the timing error was ≥2 h. To increase the therapeutic utility of early vancomycin trough concentrations, estimated true troughs can be determined by extrapolating measured values based on the time difference and CrCl.

## 1. Background

### 1.1. Vancomycin Trough Monitoring

Vancomycin is a glycopeptide antibiotic that has been used for many years to treat serious infections caused by Gram-positive pathogens, including methicillin-resistant *Staphylococcus aureus* (MRSA). Due to the changing sensitivity patterns of such organisms to vancomycin, recent dosing and monitoring guidelines include more aggressive dosing and higher target serum concentrations. The latest therapeutic guidelines call for achieving a vancomycin trough concentration of 10–15 mg/L for mild or moderate infections and 15–20 mg/L for complicated invasive MRSA infections, such as bacteremia, endocarditis, pneumonia, meningitis, and osteomyelitis [1]. Although trough concentrations constitute the primary monitoring parameter by which to evaluate the adequacy of a vancomycin dosing regimen, there is evidence to suggest that the area under the curve over 24 h (AUC_24_) is the most reliable indicator of dosing adequacy when the AUC_24_ is divided by the minimum inhibitory concentration (MIC) of the pathogen [2].

The value of AUC_24_/MIC is termed the area under the inhibitory curve (AUIC). Current guidelines suggest that an AUIC ≥400 reflects an optimal vancomycin dosing regimen [1]. Patients with a trough concentration below 15 mg/L could have AUC_24_ values that constitute adequate dosing. In such cases, increasing the dose to achieve a trough concentration of 15–20 mg/L could needlessly expose the patient to an increased risk of nephrotoxicity. Patel et al. also questioned the rationale of using the vancomycin trough concentration as a reflection of AUC_24_, concluding that the trough level is a reasonable marker of possible toxicity, but it might not be a reliable measure of therapeutic dosing [3] However, because AUC_24_ and AUIC cannot be measured directly, trough concentration monitoring continues to be recognized in the hospital setting. Rybak et al. states that “trough serum vancomycin concentrations are the most accurate and practical method for monitoring vancomycin effectiveness” [1].

### 1.2. Importance of Trough Timing

The most significant source of variability related to trough concentration monitoring is the timing of the serum concentration measurement in relation to the dosing regimen. Isolated trough monitoring, in which the serum concentration is compared against a standard range, is based on the assumption that the measured serum concentration is a true trough, measured at the end of a dosing interval. Vancomycin guidelines suggest that a trough concentration should be measured just before the next dose at steady-state [1]. As a practical consideration, institutional policy might call for trough concentrations to be measured within 30 or 60 min of the next dose. Therefore, a serum concentration designated to be a trough concentration, but actually measured a few hours before the end of the dosing interval, would constitute a false representation of trough concentration. Such an overestimation could lead a clinician to mistakenly assume that the patient’s dose should be decreased or mask the need to increase the dosing regimen for a patient who is actually sub-therapeutic [4].

As an example of the prevalence of early trough concentrations, Morrison et al. retrospectively analyzed the timing of 2597 vancomycin trough concentrations measured over a 13-month period at a large academic medical center [4]. They considered a blood sample to be drawn too early when it was taken more than 2 h before the end of the dosing interval. Results indicated that 41.3% of the vancomycin serum concentrations designated to be trough concentrations, were measured more than 2 h before the true trough would have occurred. The mean of the samples drawn too early (22.1 mg/L) was significantly larger (*p* < 0.001) than the mean of the samples drawn within the appropriate time range (15.5 mg/L). More than half (53.8%) of the early samples were above the therapeutic range, compared to 26.0% of the correctly timed samples (*p* < 0.001). Morrison et al. also studied the response of clinicians based on the vancomycin serum concentration [4]. They found that when the blood samples were drawn too early to represent a legitimate trough concentration, physicians seldom altered the dosing regimen (2%) and were much more likely to order a repeat serum concentration (44%), suggesting that they assumed the reported serum concentrations to be erroneous artifacts. Repeating a trough measurement due to mistiming can cause unnecessary delays in dosing changes and can increase the cost of care.

The previous investigators in the Morrison et al. study did not consider whether the patients were likely to have been at steady-state when the serum concentrations were measured. Davis et al. reported a survey displaying the results that assessed adherence to the 2009 consensus guidelines for vancomycin dosing and monitoring [5]. The survey was completed by 181 members of the Making a Difference in Infectious Diseases Pharmacotherapy Research Network. Of the survey respondents, 88 (54%) reported that trough concentrations at their respective institutions were only sometimes measured just before the next dose at steady-state and 73 (45%) indicated that trough concentrations were always measured according to the guidelines. The authors concluded that there was room for improvement in the timing of vancomycin trough concentrations. 

Other examples of evidence showing the prevalence of early vancomycin trough concentrations were shown in Koppula et al. and Neely et al. Koppula et al. assessed 47 vancomycin trough concentrations that were above 25 mg/L to determine whether the trough was high due to improper dosing, an abnormal pharmacokinetic pattern, inaccurate estimation of renal function, or improper timing of the measurement [6]. They attributed the cause of elevated serum concentrations to the drawing of the trough blood sample too early in 10 (21%) of the cases. Neely et al. expressed concerns about the inaccuracy of trough concentrations due to timing errors [7]. Based on a review of the measured trough concentrations of 36 patients, they found that only 7 (19%) were measured within 1 h of the next scheduled dose. When the timing threshold was relaxed to 2 h, only 14 (39%) of the samples were drawn within the time range [8]. 

## 2. Objective

The purpose of this study was to identify the frequency with which vancomycin trough concentrations are measured too early in a 400-bed institution and compare the findings to what has been reported in the literature. An additional goal was to evaluate whether a method of pharmacokinetically extrapolating mistimed vancomycin trough concentrations might have clinical utility in locating sub-therapeutic outcomes.

## 3. Methods

### 3.1. Phase 1: Vancomycin Trough Review

Following approval of the institutional review board, retrospective chart reviews of patients who had undergone vancomycin serum concentration monitoring were conducted during June 2014 and December 2014, at a 400-bed hospital. Adult patients who had vancomycin trough concentrations measured during the previous 7 months were assessed for inclusion. Patient specific parameters had to be met in their entirety in order to be included in the study. Patients were excluded if any of the following data items could not be retrieved from the medical record: age, gender, height, actual weight, serum creatinine (SCr), vancomycin indication, vancomycin dosing regimen, times of vancomycin administrations, vancomycin trough concentration, and the times of vancomycin trough concentration. The patient had to be on vancomycin for at least 3 days and the trough concentration had to be measured before the fourth dose. Patients that were receiving vancomycin in 8, 12, and 24 h intervals were included for analysis. The primary outcome was to witness the frequency of early vancomycin trough concentrations and to see how many true trough concentrations were considered sub-therapeutic after pharmacokinetic extrapolation. Secondary outcomes were to measure the mean (Range) of CrCl and the timing effects on vancomycin trough concentrations ≥10 mg/L measured more than 0.5 h before the next dose.

The time of each vancomycin trough concentration was compared to the time of the next scheduled dose and then rounded off to the nearest 15-min interval. Trough concentrations measured within 0.5 h of the next dose were considered to be appropriate. The 30-min threshold was instilled per institution protocol and also suggested by the report of Ye et al., which evaluated the criteria of 12 vancomycin therapeutic drug monitoring guidelines from around the world [9]. Creatinine clearance (CrCl) was estimated using the equation of Cockcroft and Gault [10].

### 3.2. Phase 2: Pharmacokinetic Trough Extrapolation

A second phase of the study involved estimating what the “true” trough would have been for each measured trough, and defining the difference between the two values. Trough concentrations measured too early were adjusted pharmacokinetically by predicting how much the serum concentration would have fallen during the time period between when the trough was actually measured and when it should have been measured. That time interval was designated as “t”. The vancomycin elimination rate constant (K) was determined using a linear regression formula that quantifies the relationship between K and CrCl as follows: K = CrCl × 0.00083 + 0.0044 [11]. The value of the measured trough (MT) was then extrapolated to estimate the true trough (TT), using the equation: TT = MT × e^−Kt^. With this method of determining vancomycin K, it is important to use an uncorrected value of CrCl in mL/min, not one that has been corrected to a standard body surface area of 1.73 m^2^. The value of CrCl was estimated from the Cockcroft-Gault equation. Renal function reported as eGFR cannot be used in place of CrCl for this purpose. 

### 3.3. Phase 3: The Impact of Serum Concentration, Time, and CrCl

Early trough measurement results in an overestimation of the true trough concentration. Therefore, the potential impact of a timing error for an early trough that is sub-therapeutic is not likely to be significant, because the trough would also have been sub-therapeutic if measured at the correct time. In that situation, the clinician could assume that the patient’s dosing regimen needs to be increased regardless of the timing error. The greatest possibility that an early trough measurement could have a significant therapeutic impact on a patient care decision exists when the serum concentration is at least 10 mg/L. The subset of early troughs ≥10 mg/L was identified and evaluated as a separate data set. Then the early troughs must be separated by mild to moderate or complicated infections to determine if the true extrapolated trough concentrations were sub-therapeutic. To be included for sub-therapeutic analysis, the initial measured concentration had to be within the recommended range. The recommended ranges for mild/moderate infections are 10–15 mg/L and for severe/complicated infections are 15–20 mg/L. Gram positive infections were considered to be mild/moderate unless the infection was bacteremia, endocarditis, meningitis, osteomyelitis, or hospital acquired pneumonia [1]. For the secondary outcomes, the relationship between CrCl and the extent of timing error based on the difference between the measured trough and estimated true trough was evaluated. To view this relationship, the early troughs need to be separated by timing errors of <2 h and ≥2 h, and also for CrCl values of <60 mL/min or ≥60 mL/min. 

## 4. Results

A total of 143 vancomycin trough concentrations, representing 128 patients, were included in the analysis. Results shown in Table 1 indicate that 62 (43%) of the trough concentrations were drawn >0.5 h early, and of those, 28 were measured ≥2 h early. Of the 62 troughs that were measured >0.5 h early, 48 troughs were >10 mg/L. There were 21 mild/moderate infections and 27 complicated infections that made up these 48 troughs. Table 2 illustrates that 25% of these troughs were considered to be sub-therapeutic after extrapolation. 

**Figure 1:**
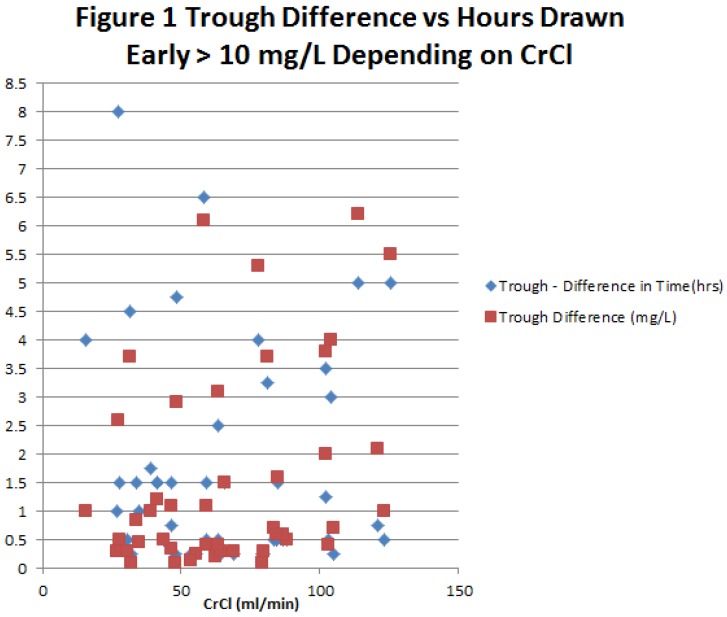
Trough Difference vs. Hours Draw Early >10 mg/L Depending on CrCl.

Results of the full analysis of the 48 early troughs that were ≥10 mg/L are shown in Table 3, with data reported based on whether CrCl was ≥60 mL/min or <60 mL/min, and whether the timing error was <2 h or ≥2 h. For each of the four subgroups, the mean and range of the measured trough are reported, along with the estimated true trough and the difference between the measured and estimated true trough. The subgroup with ≥2 h timing errors and CrCl ≥60 mL/min produced the greatest disparity between the measured and estimated true trough, with a mean of 4.6 mg/L. Based on a therapeutic range of 10–20 mg/L, 4.6 mg/L represents a relative error of 23%–46%. Table 3 also illustrates that with a CrCl <60 mL/min or a timing error <2 h, the trough error is likely to be well below 2 mg/L. This observation is further illustrated by the graphical representation of data in Figure 1. The figure displays that when a trough is taken more than 2 h early, there is a possibility that it can become sub-therapeutic regardless of renal function. 

## 5. Discussion

### 5.1. Verification of the Trough Timing Issue

The most precise trough is the concentration that is measured right before the next dose of vancomycin is administered. While being aware that the recommendation for measuring trough concentration should be within 30–60 min prior to the next scheduled dose [1], the intention of considering trough concentrations that were >0.5 h early was to investigate the frequency of sub-therapeutic trough concentrations. The collective findings of published reports are comparable to the results of this study, with 43% of troughs measured more than 0.5 h early and 19% more than 2 h early (Table 1). After extrapolating the 48 early trough concentrations that were >10 mg/L, there is a one in four chance that the true concentration could be sub-therapeutic (Table 2). This means that the dosing regimen of that 25% of early concentrations would be continued, and the patient would still be sub-therapeutic which could cause resistance, increased length of stay, and increased hospital costs. By extrapolating the early trough concentration, the pharmacist or clinician that is monitoring the patient can ensure or adjust the dose so that the regimen is therapeutic due to the linear pharmacokinetics of vancomycin. 

The effect of such timing errors could be clinically significant, based on the mean projected difference between the measured and estimated true trough of >2 mg/L in 22 (45.8%) of the 48 early trough values that were ≥10 mg/L (Table 3). For the 15 early troughs that were measured to be <10 mg/L (not listed in Table 3), the mean difference between the measured trough and estimated true trough was only 1.0 mg/L, with a maximum difference of 1.8 mg/L, despite the fact that 6 of the 15 troughs were measured at least 2 h early. Early troughs that are <10 mg/L still should be extrapolated in actual practice, in order to ensure accurate dose adjusting. 

The greatest potential for timing errors to be clinically significant is associated with trough concentrations measured at least 2 h early for a patient with a CrCl of at least 60 mg/L. As reported in Table 3, the difference between the measured and estimated true trough was at least 2.0 mg/L for all 10 values in that category, with a mean estimated true trough of 12.5 mg/L, markedly lower than the mean measured trough of 17.5 mg/L. The results in Table 3 also indicate that for the subgroup of trough concentrations measured at least 2 h early for patients with CrCl <60 mg/L, the mean difference between the measured trough and the estimated true trough was 2.1 mg/L, above the threshold of potential clinical significance established for this study. However, that observation is somewhat misleading, because the results for the category are skewed. When two extreme values are excluded, the mean difference for the remaining 10 troughs drops from 2.1 mg/L to 1.5 mg/L. To further illustrate the point, of the 12 troughs in that category, five were measured at least 5 h early and the mean timing error was 4 h. 

In general, one can assume that as the timing error increases above 2 h and/or the CrCl increases above 60 mL/min, the magnitude of the trough error increases correspondingly. Furthermore, the magnitude of error associated with a trough measured less than 2 h early could be significant if the patient’s CrCl is much greater than 60 mL/min. This is because 3 out of the 15 troughs in this group met the threshold difference of 2 mg/L. Likewise, a trough that is measured 3 or more hours early could be significant, even if a patient’s CrCl is below 60 mL/min. 

These findings suggest that the timing of vancomycin trough concentrations is an issue of relevance and importance. Pharmacists should consider evaluating the vancomycin monitoring practices of their respective institutions to determine whether a need exists to implement intervention strategies. Some efforts to improve vancomycin trough monitoring have already been reported [12,13,14,15].

### 5.2. Success of Education and Policy Interventions to Improve Trough Timing

Cardile et al. conducted a pre- and post-intervention study designed to improve the utilization of vancomycin serum concentrations [14]. Interventions included scheduling vancomycin trough blood draws apart from the “batch” of morning labs, recording blood sample collection times, and initiating pharmacist-managed therapeutic drug monitoring support. The investigators also conducted in-service training for nurses and pharmacists to emphasize the importance of measuring vancomycin trough concentrations at the proper time. Results showed a decrease in mistimed trough concentrations from the pre-intervention control group (47%) in relation to the post-intervention group (32%). They also found that trough concentrations measured during the post-intervention phase were twice as likely to be within the therapeutic range (*p* < 0.001).

Coleman and Wilson conducted a pre- and post-intervention study on the appropriateness of the timing of vancomycin trough concentrations, for which the intervention involved four voluntary educational sessions that included 114 nurses [12]. Training sessions focused on the proper use of vancomycin, the importance of blood sample timing, and the principles of serum concentration monitoring of vancomycin. Prior to training, 189 of 272 (69%) vancomycin trough concentrations were drawn within the target time frame (within 45 min of the next dose). Post-intervention results of 355 trough concentrations indicated that 74% were appropriately timed, representing a 5% improvement that was not statistically significant (*p* = 0.20). These findings suggest that education alone might not be sufficient to ensure the proper timing of vancomycin trough concentrations. 

Traugott et al. studied the effectiveness of using a computerized-prescriber-order-entry (CPOE) system to improve the appropriateness of vancomycin trough concentrations, based on a goal of measuring the trough within 1 h of the next dose [13]. After collecting baseline trough data from 100 patients, they implemented a pop-up message containing vancomycin monitoring criteria that was triggered whenever a vancomycin concentration was ordered. The CPOE system was also set to default to “predose” timing specifications when a trough concentration was ordered. Post intervention results indicated that appropriate vancomycin trough concentration measurements increased from 180 of 310 (58%) to 160 of 235 (68%), a statistically significant improvement (*p* = 0.02). The investigators concluded that CPOE strategies can be effective at improving the appropriateness of vancomycin trough monitoring, but noted that the improvement was modest, achieving only a 68% rate of compliance [13]. They suggested that prescriber acknowledgement of having reviewed the vancomycin monitoring pop-up message, along with nursing education, could lead to greater compliance rates. 

Melanson et al. evaluated the effectiveness of an information-technology-based intervention to improve the timing of vancomycin trough concentrations at the same institution involved in the study reported by Morrison et al. [5,14]. They proposed that one root cause of early trough measurement was a lack of knowledge about the importance of serum concentration timing. Prior to the study, the hospital’s computer system alerted the clinician placing a vancomycin serum concentration order that trough concentrations should be collected 60 min prior to the next dose. The new intervention involved notifying nurses of the policy for the timing of vancomycin trough concentrations and requiring acknowledgement by having the nurse check a box to indicate that the information had been reviewed. Results from 9899 vancomycin trough concentrations indicated that the percentage of timing errors dropped from 39% pre-intervention to 32% post-intervention, but the difference was not found to be statistically significant (*p* = 0.64) [14].

Interviews of 40 nurses indicated that only 33% followed the instructions for timing vancomycin serum concentrations, despite having checked off that they read the instructions in the electronic medical record. Of the 20 nurses who scheduled a serum concentration to be drawn too early, 15 (75%) reported that they scheduled the blood draw sooner in order to receive the lab result before the next scheduled dose. The investigators concluded that educational reminders alone are not sufficient to ensure proper vancomycin serum concentration timing and discovered that many nurses believe that drawing blood for a vancomycin trough concentration too early (before 2 h prior to the next dose) was not a problem [14]. Confounding the issue further was the belief of some nurses that it was acceptable to draw blood for vancomycin serum concentrations during phlebotomists’ morning rounds, regardless of how that time compared to the times of vancomycin administration. 

Currently available evidence suggests that educational efforts, process changes, systems modifications, and computer strategies produce equivocal improvement in vancomycin trough measurement. Although such interventions are warranted and are worthy of further study, it appears that a different strategy might be worthwhile. Consideration should be given to establishing a procedure whereby clinicians can project what the true trough would have been for a trough concentration that is measured too early, by applying standard pharmacokinetic principles.

### 5.3. Applications of a Pharmacokinetic Extrapolation Strategy

Once the trough concentration is deemed to be drawn early, the pharmacist can use this step wise extrapolation method instead of ordering a repeat level. The steps to use this method are to simply find the patient’s elimination rate, the difference in time from when the trough was collected and when it was scheduled, and then extrapolating the trough concentration with the prior calculations. When the clinical or pharmacokinetic pharmacist is presented with an early trough, they can use the patient’s CrCl to calculate the patient’s elimination rate. The formula for the vancomycin elimination rate (K) to use is K = CrCl × 0.00083 + 0.0044 [11]. Then with the difference in time intervals from the actual collection time and scheduled collection time, the clinician needs to convert the time in 0.5 h per 30 min intervals. For example, if the early trough subtraction was an hour and fifteen minutes early, the time interval (t) would be 1.25. To calculate the true trough, the pharmacist inserts this data into the equation C_true trough_ = C_trough measured_ × e^−kt^. When the inevitable mistimed trough is reported, pharmacokinetic extrapolation can render the trough concentration more useful for clinical application and may obviate the need to repeat a concentration. The application can be a clinical guide for a more accurate dose adjustment. Table 3 demonstrates that if a patient has a CrCl <60 mL/min and is <2 h early, it is more than likely that this application does not need to take place. 

## 6. Limitations

This analysis is based largely on the results from only 48 vancomycin trough concentrations from a single institution. Gaps in the documentation of trough timing complicated the data collection process, and the analysis was dependent on the accuracy of the reported times at which blood samples were drawn. The pharmacokinetic relationship between CrCl and vancomycin elimination rate constant is based on a linear regression formula derived from a single cohort [11].

Another limitation is the use of the elimination rate formula that is population based, according to Matzke. The population in this trial could have some inherent variability when applying the pharmacokinetic characteristics of a cohort to a broader population. Switching to continuous infusion instead of a routine daily regimen is another opportunity to prevent mistimed vancomycin trough concentrations [16]. Continuous infusion dosing can reasonably reach the target concentration without the requirement of ordering for vancomycin troughs. Nevertheless, similar error is encountered whenever CrCl is used as the basis for determining an initial vancomycin dosing regimen. Some patients might warrant a more comprehensive Bayesian pharmacokinetic analysis, but when vancomycin monitoring is based on the serum concentration analysis of a single trough concentration, the methods applied in this study represent a theoretical means of addressing timing errors.

## 7. Conclusions

The early measurement of vancomycin trough concentrations is a significant therapeutic issue, and intervention efforts designed to mitigate the causes of mistiming have produced modest success at best. The inaccuracy of mistimed trough concentrations is most likely to be clinically significant when the result is at least 10 mg/L, becomes sub-therapeutic, when the concentration was measured at least 2 h early, and when the patient’s CrCl is at least 60 mL/min. The magnitude of error increases as a direct function of time and CrCl, both of which need to be considered when assessing the usefulness of a mistimed vancomycin trough. To increase the therapeutic utility of an early vancomycin trough concentration, an estimate of the true trough can be determined by extrapolating the measured value using e^−Kt^, where K = CrCl × 0.00083 + 0.0044 and t is the time difference in hours. Further study into the mistiming of vancomycin trough concentrations and the effectiveness of various intervention strategies is warranted.

## Figures and Tables

**Table 1:** Timing of Vancomycin Trough Concentrations in Relation to the Next Scheduled Dose.

Trough Concentrations Suitable for Analysis	143
Trough Measured within 0.5 h of Next Dose	81 (57%)
Trough Measured >0.5 h and <2 h Prior to Next Dose	34 (24%)
Trough Measured ≥2 h Prior to Next Dose	28 (19%)

**Table 2:** Extrapolated True Concentrations of Early Vancomycin Trough Concentrations >10 mg/L.

Infection Type	Trough Range (mg/L)	Number of Troughs (48)	Number of Sub-Therapeutic True Concentrations ^b,c^	Percentage (%)
Mild/Moderate	10–15	21	6	28.5
Complicated ^a^	15–20	27	6	22.2
Total	10–20	48	12	25

^a^ Measured complicated infections had to be >15 mg/L. ^b^ Calculated true trough that was <10 mg/L for mild/moderate infections and <15 for complicated infections. ^c^ Estimate of the “true” trough based on extrapolating the measured trough to what it would have been at the time of the next scheduled dose, using the CrCl-based estimation of the elimination rate constant from Matzke [11].

**Table 3:** Mean *(Range)* of CrCl and Timing Effects on Vancomycin Trough Concentrations ≥10 mg/L Measured ≥0.5 h Before Next Dose.

	CrCl Group (ml/min)	Number of Troughs (n)	CrCl^a^ (ml/min)	Measured Trough (mg/L)	Estimated True Trough^b^ (mg/L)	Trough Difference^c^ (mg/L)
Trough Measured ≥2 h Prior to Next Dose	≥60	11	88.3 *(64–126)*	17.5 *(13.6–22.7)*	12.9 *(7.5–19.1)*	4.6 *(2.0–6.6)*
	<60	12	37.4 *(16–59)*	16.3 *(10.3–29.4)*	14.2 *(9.2–25.2)*	2.1 *(0.6–6.4)*
Trough Measured >0.5 and <2 h Prior to Next Dose	≥60	15	87.7 *(63–123)*	16.4 *(10.2–26)*	15.2 *(9.3–22.8)*	1.2 *(0.5–3.2)*
	<60	10	43.1 *(27–59)*	16.0 *(10.3–23.6)*	15.4 *(9.8–23.1)*	0.6 *(0.4–0.9)*
Total		48	65.5 *(16–126)*	16.6 *(10.2–29.4)*	14.5 *(7.5–25.2)*	2.1 *(0.4–6.6)*

^a^ CrCl estimate via the Cockcroft-Gault equation [10]; ^b^ Estimate of the “true” trough based on extrapolating the measured trough to what it would have been at the time of the next scheduled dose, using the CrCl-based estimation of elimination rate constant from Matzke [11].; ^c^ The difference between measured trough and the estimate of what the true trough would have been.
